# Increasing Students’ Publication Productivity: Could Launching a University Scientific Journal be a Catalyst?

**DOI:** 10.7759/cureus.3953

**Published:** 2019-01-24

**Authors:** Magdalena Pasarica, Monica Bailey, Juan C. Cendán

**Affiliations:** 1 Family Medicine, University of Central Florida College of Medicine, Orlando, USA; 2 Medical Education and Simulation, University of Central Florida College of Medicine, Orlando, USA

**Keywords:** innovation, medical student, evidence-based medicine, publications

## Abstract

Our institution established an online medical journal to promote publication opportunities and to foster a culture of scholarship. In two years of activity, there was an increase in the proportion of students reporting authorship of peer-reviewed publications at our institution suggesting an increase in students’ scholarly interest and output.

## Editorial

Scholarly output, measured by peer-reviewed publications, plays an important role in medical students’ success in their training and match to a medical residency. It was previously reported that enhancing student-mentor interactions may improve publication productivity [1].

At our institution, medical students are required to complete a two-year course in research, which involves completing a research project and reporting their findings in the format of a manuscript. There is no requirement to publish the findings, although highly encouraged. To promote publication opportunities and to foster a culture of scholarship, we established a medical journal opened to authors from any institution, such as medical doctors (MDs), doctor of philosophy (PhDs) in medical-related sciences and medical students (with a faculty co-author). “Flagship: Medical Scholarly Proceedings” journal was launched in January 2016 using Cureus.com as the online only platform. Cureus.com allows open-access manuscripts without publication fees; manuscripts are PubMed indexed and rigorously peer-reviewed. The journal does not have a traditional impact factor; however, there is a system for peer scoring and tracking of page views (proxy measures of impact). The oversight of the journal is conducted by one editorial team (one administrator and two editors) from our institution and one editorial team from the main journal platform (for a second layer of quality control). Manuscripts are first reviewed for formatting by the journal administrator and then for scientific merit by the two deputy editors (both with expertise and background as Associate Editors for reputable international journals). Next, they are reviewed by at least two experts in the specific file of the manuscript from which at least one is an M.D. and/or Ph.D. (either faculty at UCF, affiliated faculty or faculty at other academic institutions), followed by the deputy editors again and finally by the Cureus editors. The reviewer's comments are detailed and the editorial team works with submitting authors to improve submissions until all concerns are addressed, and the manuscript meets a standard of high quality. To accommodate for students’ scholastic mandates that may take priority, we do not set deadlines for re-submission, an approach commonly seen with other journals. In an effort to enhance the interaction between students and a publication mentor, the editorial team conducts several mentoring group sessions per year and provides in-person help for manuscript development, improvement and/or formatting. This opportunity is presented to students, faculty and residents during departmental or faculty development meetings.

In two years of activity, 67 articles have been published with a rejection rate of 16%. Reasons for rejections included low scores on novelty, priority or quality. Seventeen percent (17%) were original research, 29% were reviews and 54% were case reports. This distribution is probably due to the learners’ higher accessibility to interesting clinical cases in their clinical rotation vs. lower opportunities for completing long-term projects during the course of their training. Most publications were authored by university-affiliated faculty, with or without trainees as co-authors, although there have been some national and international submissions. The journal webpage has been viewed 107,000 times. Eighty-three (83) reviewers (most of them faculty or affiliated clinicians at our College) from 16 specialties contributed to the journal as unpaid volunteers. There was an average time of 92 days from submission to publication, with a standard deviation of 62 days. Delays were due primarily to reviewer response times. The unexpected challenges encountered in the process of accomplishing our goal and the proposed approaches to overcome these are presented in Table 1.

**Table 1:** Challenges encountered and the approaches proposed to overcome these challenges.

Challenges encountered	Proposed solutions
A lot of time and effort was spent on mentoring learners to submit high-quality manuscripts.	To develop innovative, less time-consuming ways to provide training for new authors (i.e., via screencast or online guides)
Recruiting faculty to perform reviews in a timely manner was challenging. The initial goal was to have the process from submission to publication completed in less than 30 days, which we were not able to achieve.	To identify, train and incentivize a wide breadth of reviewers for an efficient, easily sustainable review process.

The American Association of Medical Colleges Graduation Questionnaire, a yearly national survey completed by all graduating medical students in the United States, showed an increase in the proportion of students reporting authorship of published peer-reviewed papers at our institution (from 48.7% in 2015 to 53.8% in 2016, 68.8% in 2017 and 70.5% in 2018). These results suggest that an institution-sponsored opportunity for mentored and flexible scholarly submissions may successfully increase students’ scholarly interest and output, and faculty publication opportunity. It also provides best practices for institutions considering starting a journal for the benefit of its learners. This innovative idea for improving scholarly output of medical students could be implemented by other undergraduate and graduate medical programs. This innovative idea for improving scholarly output of medical students could be implemented by other undergraduate and graduate medical programs.
